# Infection and Isolation

**Published:** 1905-06-10

**Authors:** 


### The Hospital.

A Journal of

The Medical Sciences and Hospital Administration.

Vol. XXXVIII.—No. 976. SATURDAY, JUNE 10, 1905.

### Infection and Isolation.

At a recent sessional meeting of the Royal Sanitary Institute, held at Bristol, a very instructive paper was read by Dr. D. S. Davies, medical officer of health for the city, on the theory and practice of the effective use of isolation hospitals, and was followed by an interesting discussion. Bristol is now the sixth city in size in England; and in many important respects it has been a guide and pioneer in the path of sanitary reform. The first impetus in this direction was given to it by a previous Dr. Davies, who, if we are not mistaken, was the father of the gentleman of whose work we have now to speak. Dr. D. S. Davies has availed himself of his exceptional opportunities to place at least some portions of his subject in a new and very instructive light. He approaches the question of the conditions favourable to the growth and multiplication of disease-producing bacilli not so much from the point of view of "infection" as from that of an agriculturist dealing with the circumstances favourable or unfavourable to a particular crop, and thus assists us to remember not only that we are dealing with distinct vegetable organisms, but also that the requirements of the small-pox bacillus may differ as much from those of the diphtheria bacillus as the requirements of wheat differ from those of turnips. There has, until now, been too much tendency to lose sight of such differences in the contemplation of resemblances, and one effect of this has been the misinterpretation of a good deal of experience, with consequent failures to grasp the lessons which the facts were calculated to teach. In relation to small-pox Dr. Davies reminds us that its parasitic cause is so highly organised as to be selective in regard to the soil in which it grows, so that it cannot flourish in one which is either unsuitable or exhausted. The soil can be rendered unsuitable by vaccinia; and then the person exposed to infection neither contracts small-pox nor carries it, the infection dies out. If the soil be suitable, the disease runs its acute course, after which the soil is exhausted, and the disease cannot persist in the patient. Hence there are no chronic cases and no return cases, and a patient either definitely has or has not small-pox at any given time. He never merely carries it on his living tissues in an inert but, to others, potentially infective form. The disease, although of high infectivity, is not so, except to intimate contacts, for the first two days; and, owing to these points in its natural history, small-pox, when taken in hand upon its first introduction, allows all the four essential conditions of isolation to be fulfilled. These are (1) that the patient has not handed on his infection to another before his seclusion commenced; (2) that he has either left behind no infected material or, if he has, that it has been rendered sterile; (3) that he is prevented during the whole of his illness and convalescence from direct or indirect communication of his disease to others; and (4) that he perfectly recovers before he is cleansed and discharged from seclusion. Hence hospital isolation is generally successful in this disease, and its methods and value admit of demonstration.

In the case of diphtheria, the conditions are essentially different, except in the one circumstance that the disease is transmissible from person to person. The bacillus appears to be of more lowly organisation and is not selective as to its soil. Most or all soils prove suitable; it will grow in some form or other upon most mucous surfaces, or upon the raw skin surfaces of most children. Of the persons who receive the organism about nine-tenths become infected, in the sense that they retain and grow it, but only about half of them are susceptible to intoxication by the fungus (*i.e.* suffer from clinical diphtheria); while the remainder merely carry it, showing no symptoms or very slight symptoms themselves, but being potentially highly infectious to others. An exhausted soil forms no bar to further growth, the patient who has passed through the clinical disease recovers, but the fungus, although it can harm him no farther, may continue to flourish upon his person, having perchance gained access to the recesses of the nasal cavity, to the antrum, or to the frontal sinuses. We are thus confronted with an acute infectious disease which may become chronic and may persist for months or years, or in respect of which a patient may present any one of three conditions. He may be suffering from it; not suffering from it; carrying it. The conditions of effective isolation are obviously much more difficult to establish and to maintain than in the case of small-pox, and the failures or successes in the one case may throw little or no light upon the conditions to be fulfilled in relation to the other.

The discussion which followed upon the reading of Dr. Davies's paper was of great value inasmuch as it served to direct attention to the distinct individuality of different infective organisms, to the totally different conditions which have to be encountered in dealing with them and to the frequent inapplicability of the lessons taught by one kind of epidemic to the conditions which may be most operative in another. We all know the eagerness of the amateur "sanitarian" to discover an "open drain" or a "bad smell" in any prevalence of infective disease, whatever may be its nature. Dr. Davies tells us that the spread of diphtheria is a purely personal matter, and that in an ordinarily well looked after town insanitary conditions are inoperative with regard to it The diphtheria bacillus is too delicate an organism to live in drains or in filth heaps, and he believes that even rampant insanitary conditions would have no other effect in relation to it than to produce a somewhat susceptible mucous surface for the reception of the germ derived from another person.

